# Evidence of Acute Mycoplasma Infection in a Patient with Incomplete and Atypical Kawasaki Disease: A Case Report

**DOI:** 10.1155/2011/606920

**Published:** 2011-12-10

**Authors:** M. Ebrahim, M. Gabay, R. F. Rivas-Chacon

**Affiliations:** ^1^Miami Children's Hospital, Miami, FL 33155, USA; ^2^Department of Pediatrics, Nova Southeastern University, Fort Lauderdale-Davie, FL 33314-7796, USA; ^3^Department of Rheumatology, University of Miami, Miami, FL 33136, USA

## Abstract

The etiology of Kawasaki disease remains unknown despite extensive studies. Some researchers suggest that it is caused by an infectious agent. This is a case report where a patient with incomplete Kawasaki disease was found to have evidence compatible with acute *Mycoplasma pneumoniae* infection. This is one of the several case reports linking *Mycoplasma pneumoniae* to Kawasaki disease as a possible trigger. This is perhaps due to a superantigen or is mediated by some other mechanism. Accurate and timely testing for *Mycoplasma infections* is difficult and has its limitations. Despite this, *Mycoplasma pneumoniae* should be considered in the differential and workup for Kawasaki disease.

## 1. Case

A previously healthy, fully vaccinated 12-year-old Hispanic male presented with a 6-day history of high-grade and intermittent daily fever (up to 105°F) upon admission. His initial symptoms were sore throat, nausea, and vomiting in addition to fever. Three days prior to admission, the patient was seen in a pediatric emergency department and discharged with a diagnosis of viral illness. The fever persisted, and he became more ill appearing with complaints of weakness, malaise, and myalgias, and a faint generalized, nonpruritic rash began to appear. There was no history of joint involvement, sick contacts, recent travel, or exposures other than a family dog. Physical examination at the time of admission revealed a tachycardic (heart rate of 118 beats/min), tachypneic (respiratory rate of 20/min), febrile (101.5°F), ill-appearing patient with normal blood pressure (121/75). An erythematous macular rash was present on the face, abdomen, and extremities. He had conjunctival injection and pharyngeal erythema without observed oral ulcers. Small bilateral cervical lymphadenopathy were palpated and measured to be less than 1.5 cm in size. There was no associated edema, erythema, or desquamation of the hands or feet.

Laboratory workup was initiated for suspected infectious or rheumatologic causes. Abnormal laboratory findings included a leukocytosis (17 × 10^3^/mm^3^) with predominant neutrophils and a normocytic, normochromic anemia (12.2 mg/dL). Complete blood count showed an initial thrombocytopenia (143 × 10^3^/mm^3^) that was later followed by thrombocytosis (398 × 10^3^/mm^3^). In addition, analysis revealed an elevated CRP (14.2 mg/L), ESR (64 mm/h), and hypoalbuminemia (2.5 g/dL). *Mycoplasma pneumoniae* IgM serology was positive (Mycoplasma IgM titer ≥1.10). Chest X-ray showed faint bilateral interstitial markings without lung consolidation or collapse. Comprehensive viral studies and cultures from the throat, urine, and blood were negative. The remainder of the initial workup, which included electrolytes, urinalysis, renal function test, and lupus analyzer, was within normal range.

A diagnosis of viral syndrome was initially suspected, although a course of azithromycin (10 mg/kg/day) was started. Acute *Mycoplasma pneumoniae* infection was made after the Mycoplasma IgM serology came back positive. Subsequently, the possibility of Incomplete Kawasaki disease was entertained given the patient's presentation. This was later confirmed on day 1 of hospitalization by an echocardiogram showing mild dilatation of right coronary artery. The patient was immediately started on an IVIG (2 gm/kg) infusion and high-dose aspirin (20 mg/kg/dose every 6 hrs).

The patient continued to spike fevers for three days after IVIG infusion. A second dose of 1 gm/kg IVIG infusion was given, but the inflammatory markers remained elevated and the patient continued to spike fevers throughout. A repeat echocardiogram on day 6 of hospitalization showed progression of the disease with the addition of left main and proximal left anterior descending artery ectasia. Because of the apparent progression of the disease, pulse therapy with IV methylprednisolone (30 mg/kg) was administered. The patient's symptoms improved significantly with resolution of the fever. The patient was discharged on day 9 of hospitalization with aspirin and a prednisone tapering regimen (Table 1).

Five days after discharge from the hospital, the patient was readmitted due to recurrence of the fever, malaise, and generalized erythematous rash without any new attributable exposure. Inflammatory markers were elevated, but improved when compared to previous results. Prior discharge medications, aspirin and prednisone, were continued, and an infliximab infusion (5 mg/kg) was initiated. The patient remained febrile, and a second IV methylprednisolone pulse dose (30 mg/kg) was given after which the patient defervesced and improved. The patient was discharged home to continue a prednisone tapering and aspirin regimen (Table 2).

The patient was then followed in the outpatient setting and has had no recurrence of symptoms. A repeat echocardiogram 10 days after discharge showed the diameter of the right coronary artery to be in the upper limits of normal, but the left main and descending arteries were more dilated and prominent. A one-month follow-up echocardiogram showed complete resolution of the coronary arteries. Repeat Mycoplasma IgM serology drawn ten months after discharge was negative (Mycoplasma IgM titer ≤0.90).

## 2. Discussion

Kawasaki disease (KD) is an acute, self-limiting vasculitis of unknown etiology that usually occurs in childhood between the ages of two to five years old [1]. When untreated, 15 to 25% of patients develop coronary artery aneurysms [2]. It is diagnosed according to the clinical criteria developed by Kawasaki [3], as shown in Table 3. Some patients do not fulfill the clinical criteria for classic KD and are diagnosed based on echocardiogram findings. Such patients are diagnosed with “incomplete” KD. Such patients are usually at extreme of ages and are at more risk for developing coronary artery disease. They may also have unusual findings and manifestations such as thrombocytopenia and hypoalbuminemia.

Because of the prolonged history of unexplained fever, rash, bilateral conjunctival injection, and pharyngeal erythema, incomplete Kawasaki disease was considered in the case patient. Bilateral lymphadenopathy was present but no cervical lymph node measured 1.5 cm or greater. The patient presented with three out of the five principal diagnostic criteria along with prolonged fever characteristic of classic Kawasaki disease (Table 3). The diagnosis was confirmed sonographically with involvement of the coronary arteries.

Many characteristics of Kawasaki disease suggest that it is caused by an infectious process such as its self-limited, generally nonrecurring nature, fever, and localized outbreaks [2]. It is hypothesized that the vasculitis in KD may be triggered by an immune response to a superantigen (SAg) from an infectious pathogen in those who are genetically susceptible [4]. Numerous studies showed a skewed T-cell repertoire in KD patients, and animal models have demonstrated hallmarks of superantigen-mediated response. Many of the infectious agents linked to KD such as Staphylococci species share the presence of superantigenic activity [5].

There is limited evidence that *Mycoplasma species* might be a trigger pathogen, or at least one of them. It has been reported on several occasions that MP infection preceded classic KD [6]. MP has been reported to cause cutaneous vasculitis by immune-complex-mediated mechanisms [7]. Another species of the Mycoplasma genome, *Mycoplasma arthritidis*, has been shown to produce a superantigen suggesting the possibility that other *Mycoplasma species* may do likewise [8].

The patient had serologic evidence of acute MP infection. To the best knowledge of the writers, this is the first case report of MP infection, or at least evidence of acute infection, followed by incomplete KD.

Diagnosing MP is a difficult task, especially in the face of Kawasaki disease. It is hampered by lack of standardized, rapid, and specific methods. Laboratory tests for MP include culture, serology, and PCR. There is a high ratio of asymptomatic respiratory carriers, and because of continued carriage of the organism after infection and treatment, PCR and cultures from respiratory tract specimens carry the risk of detecting healthy carriers. In recent years, the diagnosis of MP has relied mostly on serological methods.

Given the simplicity and benign treatment for MP infection, many clinicians use single-point Mycoplasma IgM serology testing to determine treatment instead of the “gold standard” paired sera conversion test. IgM antibodies are usually produced acutely, and raised IgM levels indicate acute infection. However, it was observed that elevated levels of Mycoplasma IgM might persist for several years raising concern for false-positive results [9]. When only one is positive, antibiotics are generally given as treatment, however, a second sample is ideally required to confirm. In this patient, repeat anti-Mycoplasma level IgM test ten months after initial diagnosis was negative favoring a true-positive initial test.

MP infection can present similarly to KD with prolonged fever, rash, conjunctivitis, and lymphadenopathy, and certain species of Mycoplasma genome are known to form superantigen. In this patient, initial Mycoplasma IgM titer at presentation was positive and repeat Mycoplasma IgM titer serology was negative, favoring a true positive test at initial KD presentation. To elucidate a true relationship, if any, between Mycoplasma infection and KD, Mycoplasma testing should be considered in patients found to have KD when clinically suspected which may ultimately affect severity of disease, prognosis, and treatment. The type of testing to pursue to determine acute Mycoplasma infection in the face of KD, however, remains a dilemma.

## Figures and Tables

**Table 1:** First hospital admission inpatient course.

	Day −3	Day 0	Day 1	Day 4	Day 6	Day 9
Symptoms/signs	Vomiting, ST^∗1^, fever, malaise	Fever/rash	Fever/rash	Afebrile	Fever	Afebrile/DC^∗7^
WBC count, ×10^3^ cells/*μ*L	7.7	10.4	9.3	16.8		
Hemoglobin, g/dL	12.2	12.4	10.4	8.9		
Platelet count, ×10^3^ platelets/*μ*L	147	243	256	398		
ESR, mm/h		64		145	92	
CRP, mg/dL		14.2	10.1	7.4	4.3	
Mycoplasma IgM		Positive				
Other	RST Neg^∗2^/MST Neg^∗3^	Albumin 2.5	Bld cx-neg^∗4^	Bld cx-neg		
Echo			Dilatation of RCA^∗5^		New ectasia	
Treatment	Tylenol	Azithromycin	IVIG and ASA^∗6^	2nd IVIG/ASA	Prednisone/ASA	ASA and prednisone tapering

^∗1^sore throat/^∗2^rapid strept test negative/^∗3^mononucleosis spot test negative/^∗4^blood culture negative/^∗5^right coronary artery/^∗6^aspirin/^∗7^discharge.

**Table 2:** Second admission inpatient hospital course.

	Day 0	Day 1	Day 3	Day 4	F/U^∗5^	F/U^∗6^
Symptoms/signs	Fever/rash/malaise	Fever	Fever	Afebrile/DC^∗3^	Asx^∗4^	Asx
WBC count, ×10^3^ cells/*μ*L	14				5.2	
Hemoglobin, g/dL	8.9				12.5	
Platelet count, ×10^3^ platelets/*μ*L	350				253	
ESR, mm/h	115	108		124	31	
CRP, mg/dL	1.4	3.3		1.7	<0.5	
Mycoplasma IgM						Negative
Other	Albumin 2.5	Bld cx-neg^∗2^	Bld cx-neg			
Echo					Improved RCA^∗7^ diameter, but more dilated LMCA^∗8^	Normal
Treatment	ASA^∗1^	Prednisone/ASA	Infliximab/ASA	ASA and prednisone tapering	ASA	None

^∗1^aspirin/^∗2^blood culture negative/^∗3^discharge/^∗4^asymptomatic/^∗5^followup after 10 days from discharge/^∗6^followup after 10 months from discharge/^∗7^right coronary artery/^∗8^left main coronary artery.

**Table 3:** Classic Kawasaki disease clinical diagnostic criteria.

Clinical criteria	What did the case patient have?
Fever for ≥5 days plus 4 of the following must be present to make a definitive diagnosis:	No (patient presented with fever for ≥5 with only 3 of the following)
Polymorphous rash	Yes
Bilateral conjunctival injection	Yes
At least one of the following:	
(i) Erythema or fissuring of the lips	
(ii) Strawberry tongue	Yes
(iii) Diffuse injection of oral and pharyngeal mucosa	
Acute, nonpurulent cervical lymphadenopathy (at least one node ≥1.5 cm)	No
At least one of the following:	
(i) Erythema of palms and soles	
(ii) Indurative edema of hands and feet	No
(iii) Membranous desquamation from fingertips	
